# Modifiable health-factor burden and stroke outcomes across three population databases: cross-sectional and longitudinal evidence from NHANES, CHARLS and HRS

**DOI:** 10.21203/rs.3.rs-10727783/v1

**Published:** 2026-10-05

**Authors:** Qingliang Han, Wenliu Huang, Xianqiong Tan, Lingdang Zhang, Qinglin Feng, Wenwu He

**Affiliations:** People’s Hospital Affiliated to Chongqing Three Gorges Medical College; Chongqing University Three Gorges Hospital; Chongqing University Three Gorges Hospital; Chongqing University Three Gorges Hospital; Chongqing University Three Gorges Hospital; People’s Hospital Affiliated to Chongqing Three Gorges Medical College

**Keywords:** stroke, modifiable risk factors, brain-health score, NHANES, CHARLS, HRS, external validation

## Abstract

**Background:**

Modifiable vascular and lifestyle factors often cluster in middle-aged and older adults. We examined whether a simple harmonized health profile provides comparable stroke-related information across public population databases.

**Methods:**

We used NHANES 2007–2018, CHARLS 2018 and HRS 2018 for cross-sectional analyses, and CHARLS 2011–2018 and HRS 2014–2022 for longitudinal validation. The cross-sectional outcome was self-reported physician-diagnosed stroke history. The main exposure was a six-factor burden score covering physical inactivity, hypertension, diabetes, dyslipidemia or high cholesterol, heart disease and current smoking. Database-specific weighted logistic regression estimates were pooled using random-effects meta-analysis. Longitudinal analyses used person-wave discrete-time survival models. Prediction analyses used CHARLS for model development and HRS for external validation.

**Results:**

The adjusted cross-sectional models included 14,475 NHANES participants, 16,070 CHARLS participants and 8,175 HRS participants. Stroke history increased monotonically across burden categories in all three databases. Each additional unfavorable factor was associated with higher odds of stroke history in NHANES (odds ratio, 1.69; 95% confidence interval, 1.55–1.86), CHARLS (1.67; 1.58–1.77) and HRS (1.63; 1.52–1.76). The pooled odds ratio was 1.66 (95% confidence interval, 1.60–1.73), with little observed heterogeneity. In longitudinal models, each 1-point higher brain-health score was associated with lower hazards of incident self-reported stroke in CHARLS (hazard ratio, 0.740; 95% confidence interval, 0.712–0.769) and HRS (0.830; 0.789–0.874). The brain-health score showed moderate discrimination in CHARLS (area under the curve, 0.645) and HRS (0.648).

**Conclusions:**

A higher burden of unfavorable modifiable health factors was consistently associated with stroke history across three public datasets. Longitudinal analyses linked a higher brain-health score to lower incident self-reported stroke hazards. These profiles may support low-cost population risk stratification, but they do not support causal or individual-level clinical interpretation.

## Background

Stroke remains a leading cause of death and disability worldwide. A large proportion of this burden is attributable to modifiable behavioral, metabolic and cardiovascular factors [1, 2]. Global burden studies and the INTERSTROKE study have identified hypertension, smoking, abnormal glucose metabolism, dyslipidemia, physical inactivity and cardiovascular comorbidity as major contributors to stroke risk [1–3]. Population-based studies in China have also shown a high stroke burden with substantial clustering of vascular risk factors [4, 5]. Because these factors rarely occur in isolation, cumulative health profiles may be useful for population risk stratification.

Recent work has shifted from single risk factors toward composite health scores, including Life’s Essential 8 and the Brain Care Score [6–8]. These scores translate broad prevention concepts into measurable population-level indicators. However, much of the existing evidence comes from single cohorts or country-specific populations. The unresolved question is not whether the individual factors matter. Rather, it is whether simplified and harmonized modifiable health profiles behave consistently across public datasets that differ in geography, sampling design and variable measurement.

We therefore integrated three public databases: the US National Health and Nutrition Examination Survey (NHANES), the China Health and Retirement Longitudinal Study (CHARLS) and the US Health and Retirement Study (HRS) [9–13]. The primary aim was to evaluate whether a harmonized six-component unfavorable health-factor burden was consistently associated with self-reported stroke history (prevalent stroke). The secondary aim was to use the longitudinal structure of CHARLS and HRS to examine a nine-component modifiable brain-health score in relation to incident self-reported stroke. Prediction analyses were included to evaluate population-level risk stratification and cross-population transportability, not to develop a stand-alone clinical tool.

## Methods

### Data sources and study population

NHANES is a continuous, nationally representative survey of the noninstitutionalized US population with a complex multistage probability sampling design [9, 10]. We combined six cycles from 2007–2008 through 2017–2018. CHARLS is a nationally representative longitudinal survey of middle-aged and older adults in China [11]. The cross-sectional analysis used the 2018 wave, and the longitudinal analysis used the 2011, 2013, 2015 and 2018 waves. HRS is a longitudinal survey of US adults aged 50 years or older [12]. It is sponsored by the National Institute on Aging (grant number U01AG009740) and conducted by the University of Michigan [13]. We used RAND HRS Fat Files for health variables and the RAND HRS Tracker 2022 file for longitudinal linkage, respondent weights and public design variables [13].

All three datasets are de-identified public-use research datasets, although CHARLS and HRS require registered data access. The original survey protocols were approved by the relevant institutional review boards, and participants provided informed consent.

For cross-sectional analyses, participants aged 50 years or older with information on stroke status were eligible. For longitudinal analyses, CHARLS participants were required to be stroke-free at the 2011 baseline and to have ascertainable stroke status in at least one follow-up wave from 2013 to 2018. HRS participants were required to be stroke-free in 2014 and to have ascertainable stroke status in at least one follow-up wave from 2016 to 2022.

### Outcome definitions

The cross-sectional outcome was self-reported physician-diagnosed stroke history (prevalent stroke). In NHANES, this was defined using MCQ160F. In CHARLS and HRS, stroke history was defined using harmonized self-reported physician diagnosis variables. In longitudinal CHARLS and HRS analyses, incident self-reported stroke was identified from wave-level records as no stroke at baseline followed by the first self-reported stroke during follow-up. We use cautious language throughout because the outcomes were self-reported and not adjudicated by imaging or medical record review [14].

### Exposures

The main cross-sectional exposure was the number of unfavorable modifiable health factors, ranging from 0 to 6. Components were physical inactivity, hypertension, diabetes, dyslipidemia/high cholesterol, heart disease and current smoking. The score was modeled continuously per additional unfavorable factor and categorically as 0–1, 2, 3 and > = 4 factors.

For longitudinal analyses, we constructed a nine-component modifiable brain-health score. Favorable components included meeting physical activity recommendations, no hypertension, no diabetes, no dyslipidemia/high cholesterol, no heart disease, favorable body mass index, non-current smoking, low-risk drinking and absence of frequent restless sleep or sleep disturbance. Higher scores indicated a more favorable modifiable brain-health profile.

The six-component and nine-component scores were designed for different analytic purposes. The six-component burden score was used in cross-sectional analyses because these components could be harmonized across all three databases, including NHANES. The nine-component score was restricted to CHARLS and HRS longitudinal analyses because repeated-wave information on body size, alcohol use and sleep was available in these two cohorts. We therefore interpreted the two scores as related but not identical measures of modifiable health.

Detailed harmonized variable definitions for NHANES, CHARLS and HRS are provided in Supplementary Table S1.

### Statistical analysis

NHANES analyses accounted for sampling weights, primary sampling units and strata according to analytic guidelines [10]. The 12-year MEC weight was calculated as WTMEC2YR divided by six. CHARLS 2018 cross-sectional analyses used the wave-4 individual nonresponse adjusted weight. HRS 2018 analyses used the RAND HRS Tracker respondent weight and public design variables. Logistic regression was used to estimate ORs and 95% CIs. NHANES models were adjusted for age, sex, race/ethnicity, education, family income-to-poverty ratio and survey cycle. CHARLS and HRS models were adjusted for age, sex and education.

Database-specific estimates were combined using inverse-variance random-effects meta-analysis. Heterogeneity was evaluated using Cochran’s Q test and |^2. Subgroup analyses were conducted by sex and age group. Sensitivity analysis repeated the main burden model after excluding current smoking from the burden score.

For longitudinal validation, we restructured CHARLS and HRS into person-wave datasets. CHARLS contributed three risk intervals: 2011–2013, 2013–2015 and 2015–2018. HRS contributed four intervals: 2014–2016, 2016–2018, 2018–2020 and 2020–2022. Participants entered the longitudinal analysis if they were stroke-free at baseline and had ascertainable stroke status in at least one follow-up wave. Person-wave records were retained until the first self-reported stroke or the last observed non-stroke wave. The primary longitudinal model used a complementary log-log link with interval fixed effects and adjustment for age, sex and education. Exponentiated coefficients were interpreted as discrete-time hazard ratios. CHARLS longitudinal models used the baseline respondent weight. HRS models used the baseline respondent weight with SECU and STRATUM in survey-weighted models.

Because exact stroke-onset dates were not consistently available, Cox proportional hazards models using approximated event times and discrete-time logit models were conducted as sensitivity analyses rather than as primary longitudinal models.

For prediction analyses, CHARLS served as the development cohort and HRS served as the external validation cohort. We compared a basic demographic model, a moderate-to-vigorous physical activity (MVPA) model, a brain-health score model and a full modifiable-factor model. CHARLS-derived model coefficients were applied to HRS without refitting to test transportability rather than optimize fit. HRS calibration and decision-curve plots used intercept recalibration to account for population-level baseline-risk differences, whereas discrimination statistics used the original CHARLS-derived risk ranking. Discrimination was assessed using the area under the receiver operating characteristic curve (AUC) with 300-replicate nonparametric bootstrap confidence intervals. Calibration intercept, calibration slope, Brier score and decision-curve analysis across 0.5% to 15% risk thresholds were also generated [16]. The study was reported with reference to STROBE [17], and the prediction analysis was reported with reference to TRIPOD [15].

To examine the influence of missing covariate data in CHARLS and HRS, we performed multiple-imputation sensitivity analyses with five imputed datasets. Cross-sectional models imputed the six burden components and covariates before recomputing the burden score. Longitudinal models imputed baseline person-level brain-health score and covariates before refitting the discrete-time survival models. Rubin’s rules were used to pool estimates across imputations.

Additional details on harmonization, weighting, discrete-time survival modeling, Cox sensitivity analyses, multiple-imputation sensitivity analyses and prediction-model evaluation are provided in the Supplementary Methods.

## Results

### Study population

Before complete-case covariate restriction, the cross-sectional analytic datasets included 17,442 NHANES participants with 1,243 stroke cases, 17,242 CHARLS participants with 1,380 stroke cases and 16,657 HRS participants with 1,336 stroke cases. The complete-case adjusted main models included 14,475 NHANES participants, 16,070 CHARLS participants and 8,175 HRS participants. In all three databases, participants with stroke history were older and had a higher burden of unfavorable health factors than participants without stroke history (Table 1).

Database-level baseline characteristics are provided in Supplementary Table S2.

### Unfavorable factor burden and stroke history

Stroke history increased stepwise with a higher burden of unfavorable factors in all three databases. In NHANES, the prevalence was 1.75%, 3.71%, 6.79% and 14.67% for 0–1, 2, 3 and > = 4 factors, respectively. Corresponding estimates were 3.62%, 8.19%, 15.71% and 21.12% in CHARLS, and 3.29%, 7.33%, 10.73% and 18.91% in HRS (Fig. 1).

In fully adjusted models, each additional unfavorable factor was associated with higher odds of stroke history. The ORs were 1.69 (95% CI, 1.55–1.86) in NHANES, 1.67 (95% CI, 1.58–1.77) in CHARLS and 1.63 (95% CI, 1.52–1.76) in HRS. Compared with participants with 0–1 unfavorable factors, those with > = 4 factors had markedly higher odds of stroke history. The corresponding ORs were 6.32 (95% CI, 4.46–8.97) in NHANES, 6.29 (95% CI, 4.85–8.16) in CHARLS and 6.11 (95% CI, 4.45–8.38) in HRS (Table 2).

Random-effects pooling showed a pooled OR of 1.66 (95% CI, 1.60–1.73) per additional unfavorable factor. Between-database heterogeneity was not detected in this three-database analysis (|^2 = 0.0%; P for heterogeneity = 0.812). The positive association was present in both men and women and in both age strata. The pooled association was stronger among participants aged 50–64 years than among those aged > = 65 years (Fig. 2).

Full smoking-excluded sensitivity analyses, subgroup estimates, interaction tests and heterogeneity statistics are provided in Supplementary Tables S3-S6.

### Longitudinal brain-health score, survival validation and prediction performance

For the primary longitudinal validation, CHARLS and HRS were restructured into person-wave datasets. CHARLS included 15,215 baseline stroke-free participants with follow-up, contributing 39,830 person-wave records and 1,028 first self-reported stroke events. The complete main discrete-time model included 13,986 participants, 36,853 person-wave records and 953 events. HRS included 14,325 baseline stroke-free participants with follow-up, contributing 44,378 person-wave records and 777 events. The complete main HRS model included 12,540 participants, 39,230 person-wave records and 683 events (Table 3).

In discrete-time survival models with interval fixed effects, each 1-point higher brain-health score was associated with a lower hazard of incident self-reported stroke in both cohorts (Fig. 3). The adjusted HR was 0.740 (95% CI, 0.712–0.769; P < 0.001) in CHARLS and 0.830 (95% CI, 0.789–0.874; P < 0.001) in HRS. Results were similar in the CHARLS age > = 50 sensitivity analysis (HR, 0.753; 95% CI, 0.723–0.784). Discrete-time logit models gave ORs of 0.730 (95% CI, 0.701–0.761) in CHARLS and 0.828 (95% CI, 0.786–0.873) in HRS. Cox models using approximated event times gave HRs of 0.740 (95% CI, 0.703–0.780) in CHARLS and 0.833 (95% CI, 0.792–0.877) in HRS. Random-effects pooling of the raw 1-point score estimates showed substantial heterogeneity (|^2 = 91.8%; P for heterogeneity < 0.001). Database-specific estimates were therefore emphasized, and pooled longitudinal estimates were treated as supplementary.

Longitudinal sample counts and selected discrete-time model estimates are provided in Supplementary Tables S7 and S8.

Multiple-imputation sensitivity analyses in CHARLS and HRS produced estimates consistent with the complete-case analyses. For the cross-sectional six-factor burden, the OR per additional unfavorable factor was 1.67 (95% CI, 1.60–1.75) in CHARLS and 1.69 (95% CI, 1.59–1.79) in HRS. For the longitudinal brain-health score, the HR per 1-point higher score was 0.764 (95% CI, 0.727–0.803) in CHARLS and 0.831 (95% CI, 0.790–0.875) in HRS (Supplementary Table S11).

For prediction, the brain-health score model included 11,093 CHARLS participants with 777 events and 12,540 HRS participants with 683 events. The brain-health score model achieved an AUC of 0.645 (95% CI, 0.621–0.670) in CHARLS and 0.648 (95% CI, 0.622–0.676) in HRS. The full modifiable-factor model had an AUC of 0.662 (95% CI, 0.622–0.703) in CHARLS and 0.622 (95% CI, 0.562–0.688) in HRS (Figs. 4 and 5). The lower external AUC of the full model coincided with a smaller complete-case sample (3,792 CHARLS participants and 2,750 HRS participants), suggesting that the simpler score may be more robust to missing data in applied settings.

Complete prediction-model performance metrics, calibration statistics, recalibration details and available-case counts are provided in Supplementary Tables S9 and S10. Multiple-imputation sensitivity results are provided in Supplementary Table S11. Aggregate source data for the main and supplementary tables and figures are provided as supplementary source data.

## Discussion

This multi-database study evaluated whether simple modifiable health profiles carry reproducible stroke-related information across public population datasets. The cross-sectional analyses showed a consistent dose-response pattern across NHANES, CHARLS and HRS. Each additional unfavorable factor was associated with about 60% to 70% higher odds of self-reported stroke history. The pooled estimate showed little observed between-database heterogeneity, despite differences in country, survey design, age structure and variable measurement. Because only three databases were available for pooling, this finding should be interpreted as consistency within the present datasets rather than definitive evidence of universal homogeneity. It also implies limited statistical power to detect modest heterogeneity.

The principal contribution is not the identification of new stroke risk factors. Hypertension, smoking, abnormal glucose metabolism, dyslipidemia, physical inactivity and cardiovascular comorbidity are already established determinants of stroke [1–3, 18]. The contribution is a reproducibility test. We asked whether a minimal harmonized burden score could retain a similar stroke-related signal across three widely used public datasets. This is a practical question for secondary epidemiological research, where variables are often comparable but not identical. The consistency of the cross-sectional estimates suggests that cumulative unfavorable health profiles can be measured reproducibly across these data sources.

The longitudinal analyses addressed a separate question. By using the repeated-wave structure of CHARLS and HRS, we examined whether a broader modifiable brain-health score was associated with subsequent self-reported stroke. Higher scores were associated with lower discrete-time hazards in both cohorts. This temporal ordering strengthens the public-health relevance of the score, but it does not remove residual confounding. The stronger association in CHARLS than in HRS occurred alongside higher crude event intensity in CHARLS. In Cox-format descriptive summaries, CHARLS contributed 1,028 events over 96,245 person-years (10.68 per 1000 person-years), whereas HRS contributed 777 events over 90,854 person-years (8.55 per 1000 person-years). CHARLS also included a longer 2015–2018 interval, whereas HRS follow-up was organized in approximately 2-year intervals. Longer interval ascertainment may increase uncertainty about the timing and detection of events. Differences in baseline vascular risk, age distributions, missingness, health-care access and self-reported stroke ascertainment may also contribute to the observed heterogeneity.

This distinction matters for interpretation. The three-database cross-sectional analysis supports reproducible association with stroke history. The two-cohort longitudinal analysis supports directionally consistent risk stratification for incident self-reported stroke. These forms of evidence are complementary, but they are not interchangeable. We therefore emphasized database-specific longitudinal estimates when heterogeneity was high and treated the pooled longitudinal estimate as supplementary.

The prediction findings also require cautious interpretation. AUC values around 0.65 indicate moderate discrimination rather than clinical-grade prediction. This performance is still informative for a low-cost score based largely on questionnaire and self-reported variables. However, it is not sufficient for individual clinical decision-making. The score may be more useful for population screening, risk communication and identifying groups who may benefit from more intensive vascular risk assessment. External validation in HRS strengthened the analysis by testing transportability beyond the CHARLS development setting. The full modifiable-factor model did not improve external validation performance, despite using more variables. This pattern may reflect complete-case selection, overfitting to CHARLS measurement structure, and residual non-equivalence of lifestyle and clinical variables across CHARLS and HRS.

This study extends prior work on Life’s Essential 8 and the Brain Care Score in three ways [6–8]. First, it evaluates deliberately simplified profiles rather than a fully optimized clinical algorithm. Second, it separates cross-sectional reproducibility from longitudinal prediction, reducing causal overstatement from NHANES. Third, it uses HRS as an external validation population for models developed in CHARLS. This design makes the study less dependent on one dataset and more relevant to comparative population-health research.

Several limitations should be considered. First, all stroke outcomes were self-reported physician diagnoses rather than adjudicated events. This may introduce recall error, under-ascertainment or false-positive classification, especially in older participants and across settings with different access to diagnosis [14]. The datasets also did not provide consistently harmonized ischemic and hemorrhagic stroke subtypes, so subtype-specific risk patterns could not be examined. Second, complete-case analyses may have introduced selection bias. This issue was most visible in the full prediction model, which retained substantially fewer CHARLS and HRS participants than the simpler score model. Multiple-imputation sensitivity analyses in CHARLS and HRS were directionally consistent with the main estimates, but they imputed harmonized derived variables rather than the full raw item structure and did not replace the prespecified complete-case primary models. Estimates from the full prediction model should therefore be interpreted as exploratory and may have limited generalizability. Third, the NHANES component was cross-sectional and cannot establish temporality. Fourth, harmonization required simplified variable definitions. Physical activity, dyslipidemia, sleep, alcohol use and cardiometabolic conditions were measured differently across surveys, and residual measurement non-equivalence is likely. Fifth, survey weights and available design variables were used where possible, but survey structures are not fully identical across NHANES, CHARLS and HRS. Sixth, longitudinal effect sizes differed between CHARLS and HRS, and the pooled longitudinal estimate showed substantial heterogeneity. We therefore emphasize database-specific estimates and treat longitudinal pooling as supplementary. Seventh, exact stroke-onset dates were not consistently available. Discrete-time survival models were therefore used as the primary longitudinal approach, whereas Cox models relied on approximated event times and were treated as sensitivity analyses.

Future work should test whether discrimination improves when harmonized biomarkers and richer behavioral measures are available. Candidate additions include measured blood pressure, lipid and glucose biomarkers, cognitive testing, diet quality, sleep quality, social engagement, TyG, ABSI and TyG-ABSI [19–22]. Linkage to adjudicated stroke events, mortality outcomes or electronic health records would help clarify whether the score captures incident disease risk, health-system contact, or both.

## Conclusions

Across NHANES, CHARLS and HRS, a higher burden of unfavorable modifiable health factors was consistently associated with self-reported stroke history. In longitudinal CHARLS and HRS analyses, a higher modifiable brain-health score was associated with lower discrete-time hazards of incident self-reported stroke. These findings support the use of simple modifiable health profiles in stroke prevention research and population risk stratification. They do not support stand-alone clinical prediction or causal interpretation without stronger outcome validation.

## Supplementary Material

This is a list of supplementary files associated with this preprint. Click to download.

- Additionalfile1Supplementarymethodsandtables.docx

## Figures and Tables

**Figure 1:**
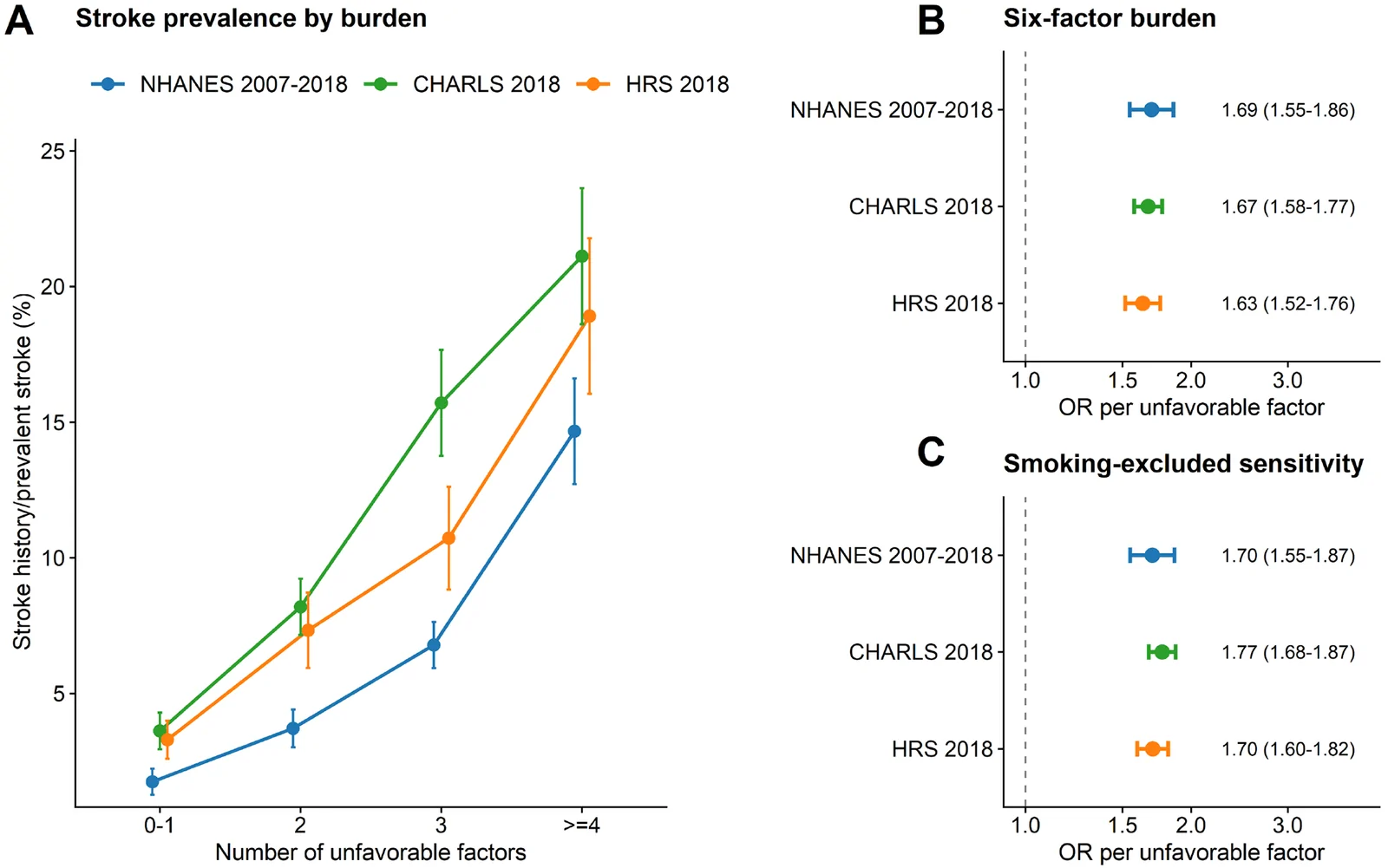
Unfavorable health-factor burden and stroke history across three databases Panel A shows the prevalence of stroke history across burden categories. Panel B shows adjusted ORs per additional unfavorable factor in the main six-factor burden model. Panel C shows the smoking-excluded sensitivity analysis. Points and error bars indicate estimates and 95% CIs. Abbreviations: CHARLS, China Health and Retirement Longitudinal Study; CI, confidence interval; HRS, Health and Retirement Study; NHANES, National Health and Nutrition Examination Survey; OR, odds ratio.

**Figure 2:**
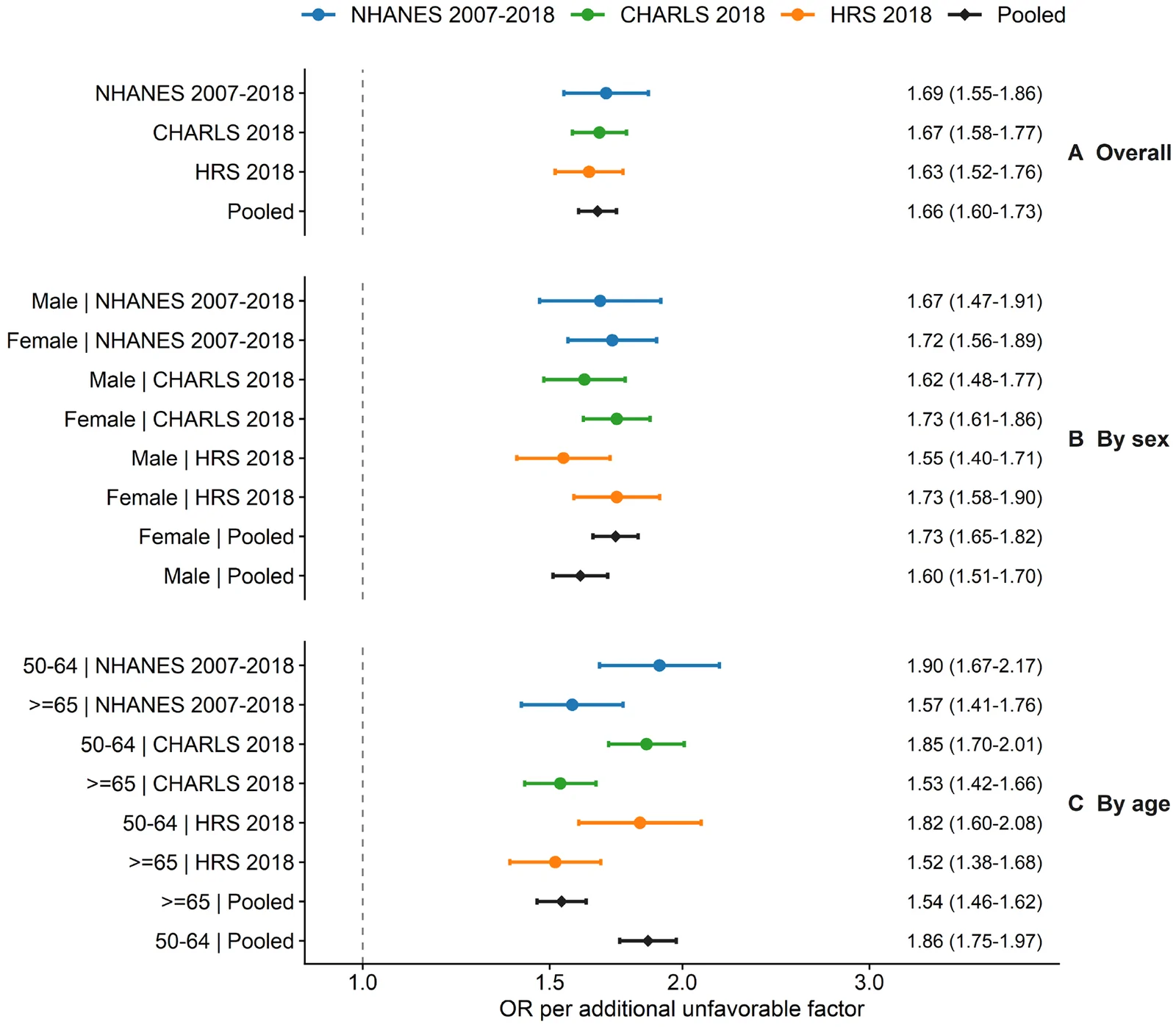
Subgroup and pooled associations of unfavorable health-factor burden with stroke history The forest plot shows database-specific and pooled estimates overall and within sex and age strata. Pooled estimates were obtained using random-effects meta-analysis. Abbreviations: CI, confidence interval; OR, odds ratio.

**Figure 3:**
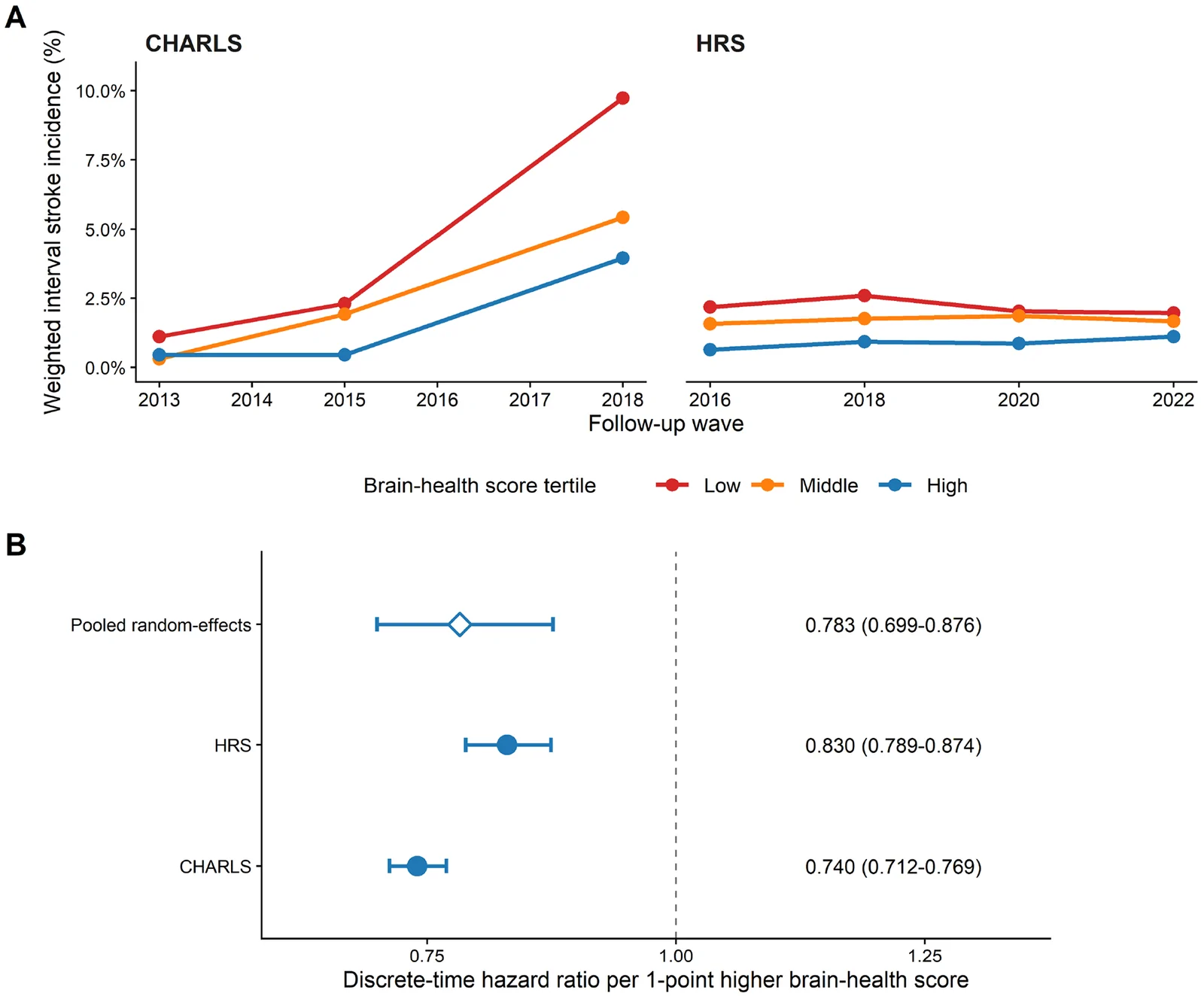
Longitudinal validation of the brain-health score in CHARLS and HRS Panel A shows weighted interval-specific incidence of incident self-reported stroke by brain-health score tertile. Panel B shows database-specific and random-effects pooled discrete-time HRs per 1-point higher brain-health score. Between-database heterogeneity for the continuous-score estimate was substantial (I^2=91.8%; P for heterogeneity<0.001). The pooled estimate is shown for reference only, and database-specific estimates should be emphasized. Abbreviations: CHARLS, China Health and Retirement Longitudinal Study; CI, confidence interval; HRS, Health and Retirement Study; HR, hazard ratio.

**Figure 4:**
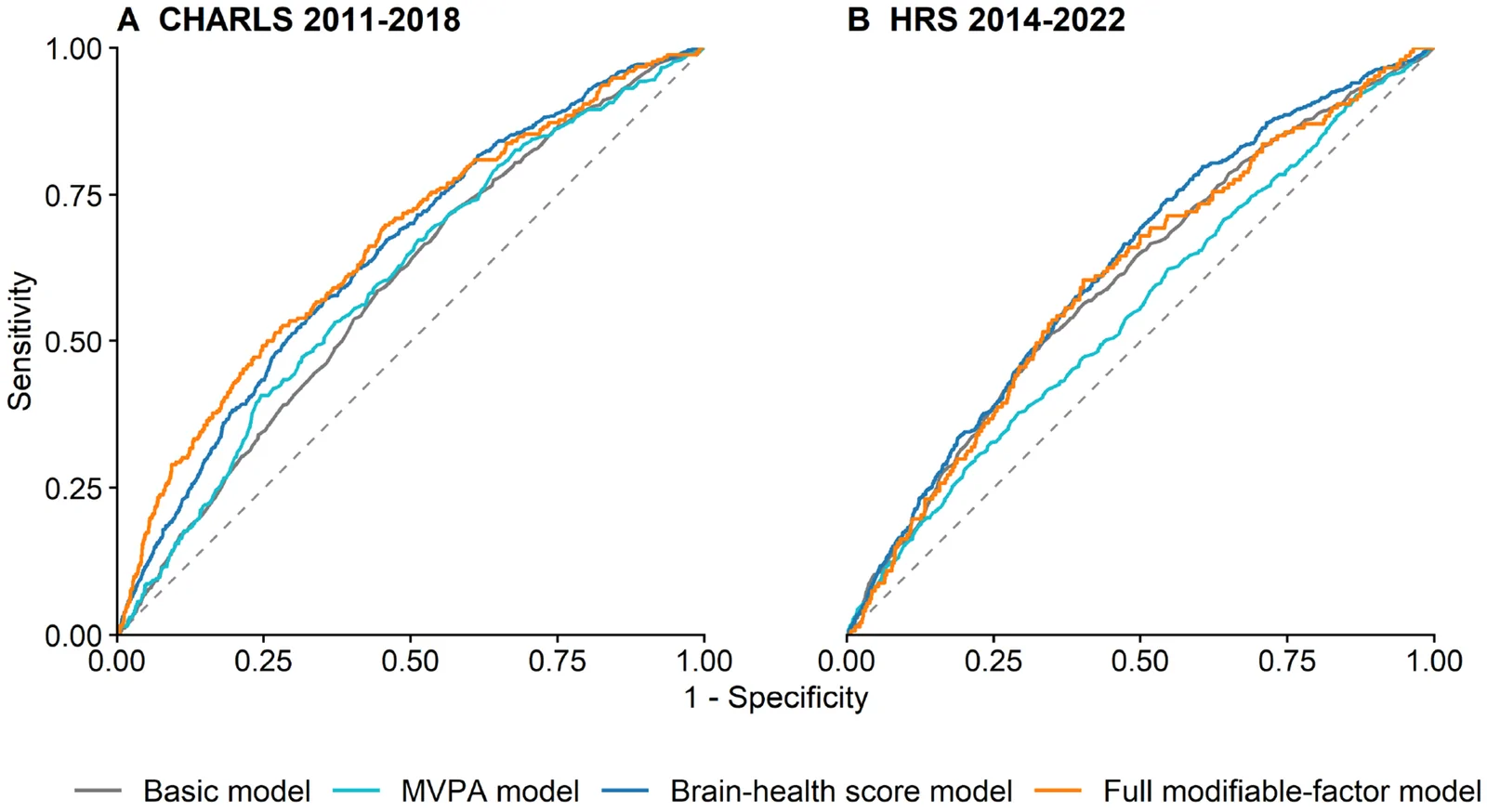
Prediction-model discrimination in CHARLS and HRS Receiver operating characteristic curves compare demographic, MVPA, brain-health score and full modifiable-factor models in CHARLS and HRS. CHARLS was used as the development cohort, and HRS was used as the external validation cohort. Abbreviations: AUC, area under the receiver operating characteristic curve; CHARLS, China Health and Retirement Longitudinal Study; HRS, Health and Retirement Study; MVPA, moderate-to-vigorous physical activity; ROC, receiver operating characteristic.

**Figure 5:**
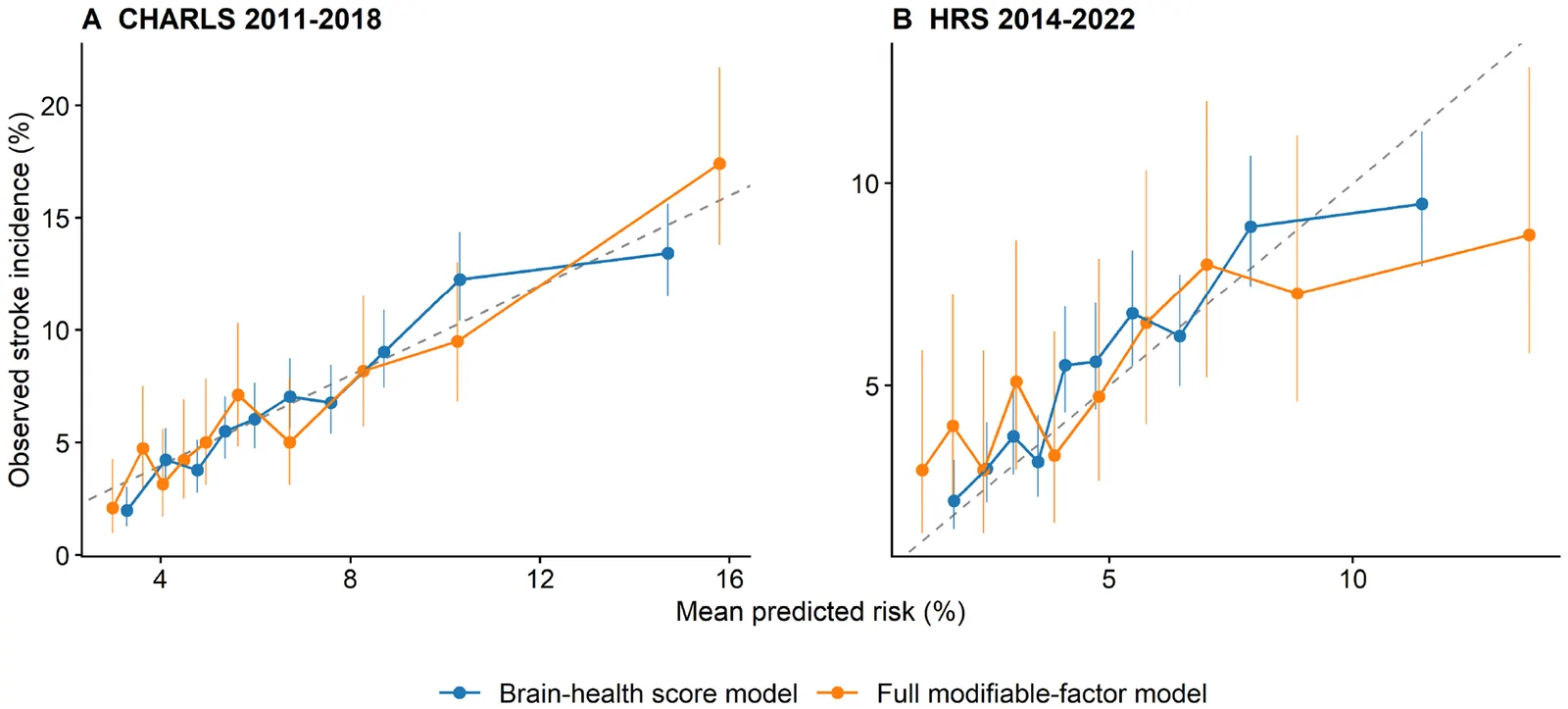
Calibration of the brain-health score and full modifiable-factor models Calibration curves compare predicted and observed risks for the brain-health score and full modifiable-factor models in CHARLS and HRS. The diagonal line represents ideal calibration. Abbreviations: CHARLS, China Health and Retirement Longitudinal Study; HRS, Health and Retirement Study.

**Table 1:** Baseline characteristics by stroke history status in the complete-case adjusted cross-sectional models

Dataset	Characteristic	No stroke	Stroke
NHANES 2007–2018	N	13,419	1,056
NHANES 2007–2018	Age, years, mean (SD)	63.0 (9.1)	68.9 (9.3)
NHANES 2007–2018	Female, %	53.4	55.1
NHANES 2007–2018	Physical inactivity, %	68.6	82.9
NHANES 2007–2018	Hypertension, %	50.0	78.6
NHANES 2007–2018	Diabetes, %	16.5	34.7
NHANES 2007–2018	Dyslipidemia/high cholesterol, %	52.2	63.9
NHANES 2007–2018	Heart disease, %	12.0	38.9
NHANES 2007–2018	Current smoking, %	14.6	19.5
NHANES 2007–2018	Unfavorable factors, mean (SD)	2.1 (1.3)	3.2 (1.3)
CHARLS 2018	N	14,775	1,295
CHARLS 2018	Age, years, mean (SD)	63.8 (9.8)	68.0 (9.6)
CHARLS 2018	Female, %	50.9	45.3
CHARLS 2018	Physical inactivity, %	41.2	61.7
CHARLS 2018	Hypertension, %	39.0	71.8
CHARLS 2018	Diabetes, %	13.2	25.2
CHARLS 2018	Dyslipidemia/high cholesterol, %	22.6	43.9
CHARLS 2018	Heart disease, %	19.4	36.6
CHARLS 2018	Current smoking, %	27.4	22.1
CHARLS 2018	Unfavorable factors, mean (SD)	1.6 (1.2)	2.6 (1.3)
HRS 2018	N	7,445	730
HRS 2018	Age, years, mean (SD)	66.1 (9.5)	69.8 (10.6)
HRS 2018	Female, %	49.4	42.8
HRS 2018	Physical inactivity, %	24.0	41.3
HRS 2018	Hypertension, %	56.6	79.7
HRS 2018	Diabetes, %	23.7	37.1
HRS 2018	Dyslipidemia/high cholesterol, %	32.4	40.4
HRS 2018	Heart disease, %	22.8	56.7
HRS 2018	Current smoking, %	22.8	23.1
HRS 2018	Unfavorable factors, mean (SD)	1.8 (1.3)	2.8 (1.3)

Note. Values are analytic complete-case counts, percentages, or mean (SD), as appropriate. Stroke refers to prevalent self-reported doctor-diagnosed stroke in NHANES and self-reported stroke history in CHARLS and HRS. CHARLS, China Health and Retirement Longitudinal Study; HRS, Health and Retirement Study; NHANES, National Health and Nutrition Examination Survey.

**Table 2:** Association between unfavorable health-factor burden and stroke history

Dataset	Analysis	n/events	OR (95% CI)	P value
NHANES 2007–2018	2 vs 0–1 factors	13,956/995	1.85 (1.26–2.70)	0.002
NHANES 2007–2018	3 vs 0–1 factors	13,956/995	2.83 (1.95–4.11)	< 0.001
NHANES 2007–2018	>=4 vs 0–1 factors	13,956/995	6.32 (4.46–8.97)	< 0.001
NHANES 2007–2018	Per additional factor	13,956/995	1.69 (1.55–1.86)	< 0.001
CHARLS 2018	2 vs 0–1 factors	16,070/1,295	2.14 (1.67–2.74)	< 0.001
CHARLS 2018	3 vs 0–1 factors	16,070/1,295	4.32 (3.33–5.60)	< 0.001
CHARLS 2018	>=4 vs 0–1 factors	16,070/1,295	6.29 (4.85–8.16)	< 0.001
CHARLS 2018	Per additional factor	16,070/1,295	1.67 (1.58–1.77)	< 0.001
HRS 2018	2 vs 0–1 factors	8,175/730	2.18 (1.56–3.05)	< 0.001
HRS 2018	3 vs 0–1 factors	8,175/730	3.20 (2.27–4.51)	< 0.001
HRS 2018	>=4 vs 0–1 factors	8,175/730	6.11 (4.45–8.38)	< 0.001
HRS 2018	Per additional factor	8,175/730	1.63 (1.52–1.76)	< 0.001
Random-effects pooled estimate	Per additional factor	3 databases	1.66 (1.60–1.73)	< 0.001

Note. Odds ratios are adjusted for age, sex and education in database-specific models. The random-effects pooled estimate summarizes the per-additional-factor association across the three databases. CI, confidence interval; OR, odds ratio.

**Table 3:** Longitudinal survival validation of the brain-health score

Analysis	CHARLS 2011–2018	HRS 2014–2022
Baseline stroke-free participants with follow-up	15,215	14,325
Person-wave records	39,830	44,378
Incident self-reported stroke events	1,028	777
Complete discrete-time model participants/events	13,986/953	12,540/683
Discrete-time HR per 1-point higher score	0.740 (0.712–0.769)	0.830 (0.789–0.874)
Discrete-time logit sensitivity, OR	0.730 (0.701–0.761)	0.828 (0.786–0.873)
Cox sensitivity, HR	0.740 (0.703–0.780)	0.833 (0.792–0.877)

Note. The discrete-time model used a complementary log-log link with interval fixed effects. The Cox model was retained as a sensitivity analysis using approximated event times. CI, confidence interval; HR, hazard ratio; OR, odds ratio.

## Data Availability

NHANES data are available from the National Center for Health Statistics. CHARLS data are available through the China Health and Retirement Longitudinal Study data portal (https://charls.charlsdata.com) after registration. HRS and RAND HRS data products are available through the Health and Retirement Study data portal and RAND HRS product pages. Aggregated source data underlying the tables and figures are provided as supplementary source data. Individual-level CHARLS and HRS data are not redistributed in this submission package because users should obtain them directly from the original data providers under their data-use conditions.

## Abbreviations

AUC: area under the receiver operating characteristic curve
BCS: Brain Care Score
BMI: body mass index
CHARLS: China Health and Retirement Longitudinal Study
CI: confidence interval
HRS: Health and Retirement Study
HR: hazard ratio
MVPA: moderate-to-vigorous physical activity
NHANES: National Health and Nutrition Examination Survey
OR: odds ratio
TRIPOD: Transparent Reporting of a multivariable prediction model for Individual Prognosis Or Diagnosis.
